# Variants of sequence family B *Thermococcus kodakaraensis* DNA polymerase with increased mismatch extension selectivity

**DOI:** 10.1371/journal.pone.0183623

**Published:** 2017-08-23

**Authors:** Claudia Huber, Andreas Marx

**Affiliations:** Department of Chemistry, University of Konstanz, Konstanz, Germany; Istituto di Genetica Molecolare, ITALY

## Abstract

Fidelity and selectivity of DNA polymerases are critical determinants for the biology of life, as well as important tools for biotechnological applications. DNA polymerases catalyze the formation of DNA strands by adding deoxynucleotides to a primer, which is complementarily bound to a template. To ensure the integrity of the genome, DNA polymerases select the correct nucleotide and further extend the nascent DNA strand. Thus, DNA polymerase fidelity is pivotal for ensuring that cells can replicate their genome with minimal error. DNA polymerases are, however, further optimized for more specific biotechnological or diagnostic applications. Here we report on the semi-rational design of mutant libraries derived by saturation mutagenesis at single sites of a 3’-5’-exonuclease deficient variant of *Thermococcus kodakaraensis* DNA polymerase (KOD pol) and the discovery for variants with enhanced mismatch extension selectivity by screening. Sites of potential interest for saturation mutagenesis were selected by their proximity to primer or template strands. The resulting libraries were screened via quantitative real-time PCR. We identified three variants with single amino acid exchanges—R501C, R606Q, and R606W—which exhibited increased mismatch extension selectivity. These variants were further characterized towards their potential in mismatch discrimination. Additionally, the identified enzymes were also able to differentiate between cytosine and 5-methylcytosine. Our results demonstrate the potential in characterizing and developing DNA polymerases for specific PCR based applications in DNA biotechnology and diagnostics.

## Introduction

DNA polymerase fidelity is crucial for all DNA dependent processes of living species [1–6]. Replicative DNA polymerases are able to polymerize a new DNA strand with intrinsic error rates as low as 10^−5^ [7–9]. This intrinsic fidelity is determined by a number of factors: initially the formation of hydrogen-bonds by correct pairings of A-T and C-G was seen as main contributing factor [10, 11], but later studies indicated that inter-nucleobase hydrogen-bonding can only be partly responsible for the DNA polymerase fidelity measured [12, 13]. The fit of the geometry of the incoming dNTP to the active site plays a crucial role in selecting the right nucleotide [2, 3, 14–17]. In addition, stabilization of the pyrophosphate leaving group and the rate of phosphoryl transfer upon dNMP incorporation are discussed [18, 19]. Additionally, fidelity is further determined by the ability of a DNA polymerase to discriminate between the extension of the nascent DNA strand from canonical and mismatched primer ends [2, 4, 20, 21]. DNA polymerases do extend mismatched primer ends, albeit with lower efficiency than the matched counterparts [3, 5, 20, 22]. Because cells have established a number of additional mechanisms to ensure genome stability—namely proofreading (i.e. excision of mismatched nucleotides)—during replication [23–25] and post-replicative mismatch repair (reviewed in [26, 27])–genome replication is carried out with as little as 10^−9^–10^−10^ errors per synthesized phosphodiester bond. Together, these strategies keep the replicative mutation rate of a cell below the background level of e.g. DNA damage, so there is no evolutionary pressure to further improve DNA polymerase fidelity.

Studies on DNA polymerase variants have contributed largely to our understanding of DNA polymerases and replication fidelity [4]. Several studies have identified DNA polymerases with decreased selectivity [28–32]. DNA polymerases with increased fidelity were also generated [33–37], as well as others with altered substrate tolerances [38–41].

Beyond the mechanistic insight obtained from these engineering approaches, DNA polymerases with increased selectivity are especially interesting, because they also pose a source for developing tools for specific biotechnological as well as diagnostic applications. Several studies were conducted to investigate the effects of amino acid exchanges on polymerase fidelity through mismatch extension selectivity of DNA polymerases from sequence family A. In *E*. *coli* DNA polymerase I and *Thermus aquaticus* DNA polymerase it has been shown that within motif C, which plays a key role in primer/template recognition [42], hydrophobic amino acid substitutions result in polymerase variants with increased mismatch extension selectivity [35, 43, 44]. Amino acid exchanges have been analyzed for the B-family DNA polymerase RB69 and the eukaryotic DNA polymerase δ in comparison to A-family members, showing furthermore that no direct comparisons can be made between analogous amino acids even within the same sequence family: exchanges that led to antimutator variants of Pol δ, turned the corresponding RB69 mutant into a strong mutator [45]. Although B family polymerases are used in many core biotechnological applications, they are still significantly less well understood and especially structural studies of the ternary complex remain elusive [46–48]. The DNA polymerase from *Thermococcus kodakaraensis* is a prominent member of sequence family B and our recent studies have shown the potential of engineering this DNA polymerase to be able to recognize mismatched base pairs [49]. Some of our previous works have shown that the combination of site specific mutagenesis and screening techniques in order to generate tailor-made enzymes to explore the scope of altered DNA polymerase selectivity for both biotechnological as well as diagnostic applications is a promising approach [37, 49–51].

In this study, we present the generation and evaluation of rationally designed libraries of a 3’-5’-exonuclease deficient variant of *Thermococcus kodakaraensis* DNA polymerase (KOD pol) and report on the influence of single amino acid exchanges at sites with direct contact to either primer or template strand on mismatch extension. We could identify several variants with increased mismatch extension selectivity that were characterized in both allele-specific amplification and methylation-specific PCR assays.

## Materials and methods

### Reagents and instruments

DNA oligonucleotides were ordered from biomers.net GmbH, dNTPs from Fermentas. Pfu turbo DNA polymerase was bought from Agilent, DpnI and T4 DNA Ligase from NEB. For PCR clean-up the NucleoSpin^®^ Gel and PCR clean-up kit from Macherey-Nagel was used according to the manufacturer’s manual. Plasmid preparations were conducted using the QIAprep Spin Miniprep Kit from QIAGEN and plasmids were sequenced at GATC Biotech AG. A 3’-5’-exonuclease deficient KOD pol (harboring aa substitutions D141A and E143A) and its respective mutants were recombinantly expressed from a pET21a vector (Novagen) in *E*. *coli* BL21 (DE3) cells and purified with complete His-Tag Purification Resin from Roche as described previously [52]. Vivaspin columns were from Sartorius, absorbance was measured with a NanoDrop 1000 Spectrophotometer from Thermo Scientific. PCR reactions were performed in a BIOER LifeECO cycler, real-time PCR was carried out in a Roche LightCycler 96 system with visualization of DNA formation via SYBR Green I from Sigma. Genomic reference DNA samples were bought from NIBSC (Prothrombin Mutation G20210A, Human gDNA, 1st International Genetic Reference Panel 2005 –WHO International Standard or Reference Reagent Product Number 05/130).

### Library construction, expression and protein purification

PCR forward primers for cloning harbored a codon mismatch to introduce one of the 19 desired mutations each. Reverse primers were 5’-phosphorylated for ligation of the PCR product. Oligonucleotides were dissolved in water to a concentration of 100 μM. 19 single PCR reactions were carried out per mutated site, in order to obtain a full library at any investigated position. After mutagenesis PCR, the template DNA was digested with the methylation sensitive endonuclease DpnI. PCR products were purified from agarose gels and ligated for either 2 h at room temperature or overnight at 16°C. Chemically competent *E*. *coli* were transformed with 5 μl of a ligation reaction and positive clones were selected via carbenicillin resistance. Single clones were picked, plasmids prepared and sequenced to ensure correct mutagenesis. Libraries were established as described before [52]. Briefly, one clone per well was grown overnight in 384-well plates, containing 150 μl LB-medium supplemented with 100 μg/ml carbenicillin per well at 37°C on a plate shaker (180 rpm). After overnight incubation, cultures were supplemented with glycerol to a final concentration of 25% and stored at -80°C. Expression of the libraries was done in 96-deep well plates, in a volume of 500 μl, inoculated from the libraries [53]. After IPTG induction, cells were further incubated for 2.5 hours and harvested by centrifugation. Cell pellets were lysed in 120 mM TrisHCl pH 8, 10 mM KCl, 6 mM (NH_4_)_2_SO_4_, 0.1% Triton, 1.5 mM MgCl_2_, 1 mM PMSF, 1 mg/ml lysozyme at 37°C for 20 min, followed by heat denaturation at 75°C for 40 min and centrifugation at 4.000 x g for 60 min. Expression levels were checked via SDS-PAGE, cleared lysates were directly used for screening. For protein purification, expression was performed in a volume of 50 ml and lysis was carried out accordingly. Cleared lysates were incubated with 50% (v/v) Ni-beads slurry at 4°C, followed by a wash step. Proteins were eluted with 100 mM TrisHCl pH 8, 3 mM MgCl_2_, 200 mM imidazole, the latter being removed subsequently by centrifuging eluates in Vivaspin columns. Purified enzymes were stored in 120 mM TrisHCl pH 8, 10 mM KCl, 6 mM (NH_4_)_2_SO_4_, 0,1% Triton, 1.5 mM MgCl_2_, 50% glycerol. Protein concentrations were determined by absorbance measurements and purified enzymes were stored at -20°C.

### Screening of the library

Initial screening reactions were done using primers to amplify a fragment spanning 92 nucleotides of the human NANOG gene and corresponding primers to generate an 86 bp long PCR product [49, 54]. Reactions consisted of 10 μl and contained 200 μM dNTPs, 100 nM forward primer harboring either G/A/T or C at its 3’end for “match” or “mismatch” case (5’-d(CTT GGT GAG ACT GGT AGA CG/A/T/C)-3’) and reverse (5’-d(TTA GAC CCA CCC CTC CTG GCG)-3’) primer respectively, 100 pM template DNA and 1x SYBR Green I in buffer (120 mM TrisHCl pH 8, 10 mM KCl, 6 mM (NH_4_)_2_SO_4_, 0.1% Triton, 1.5 mM MgCl_2_). Real time PCR experiments had an initial denaturation at 95°C for 2 min followed by amplification over 50 cycles with denaturation at 95°C for 10 s and annealing and elongation at 68°C for 20 s. Melting curves were measured immediately after PCR amplification. Two independent experiments were conducted using lysates from independent library expressions.

### Real time PCR using purified enzymes

Reactions were carried out as described above with 400 nM purified enzyme. For the prothrombin factor II single nucleotide polymorphism (SNP) primers were used amplifying a 163 bp fragment: forward primer “C-match” or “A-mismatch” (5’-d(CTG GGA GCA TTG AGG CTC/A)-3’), reverse primer (5’-d(CCG CCT GAA GAA GTG GAT AC)-3’). Detection of methylation was done using primers amplifying a 74 bp fragment of the BRCA1 gene [55]: forward primer ending in G (“match”) or A (“mismatch”): (5’-d(AAG TCT CAG CGA GCT CAC G/A)-3’) and reverse (5’-d(CGT GGC AAC GGA AAA GCG)-3’). In both cases, synthetic oligomers corresponding to the genes of interest served as template DNA. All reactions were repeated and reproduced at least three times.

### Primer extension assay

Single-nucleotide incorporation primer extension experiments were carried out followed by analysis via denaturing polyacrylamide gel electrophoresis (PAGE) and visualization by autoradiography. Reactions contained 120 mM TrisHCl pH 8, 10 mM KCl, 6 mM (NH_4_)_2_SO_4_, 0.1% Triton, 1.5 mM MgCl_2_, 150 nM purified KOD pol (wildtype or mutants). DNA primer (150 nM -labeled primer (5’-[32P]-d(CTG GGA GCA TTG AGG CTC)-3’) and 200 nM FIIG template (5’-d(AGG GGG CCA CTC ATA TTC TGG GCT CCT GG AAC CAA TCC CGT GAA AGA ATT ATT TTT GTG TTT CTA AAA CTA TGG TTC CCA ATA AAA GTG ACT CTC AGC GAG CCT CAA TGC TCC CAG TGC TAT TCA TGG GC)-3’) or FIIA (5’-d(AGG GGG CCA CTC ATA TTC TGG GCT CCT GG AAC CAA TCC CGT GAA AGA ATT ATT TTT GTG TTT CTA AAA CTA TGG TTC CCA ATA AAA GTG ACT CTC AGC AAG CCT CAA TGC TCC CAG TGC TAT TCA TGG GC)-3’) were used for match or mismatch case respectively. After initial denaturation for 2 min at 95°C, reactions mixtures were cooled to 55°C and primer extension reactions were started by addition of dGTP to a final concentration of 200 μM and proceeded at 55°C for the desired incubation times. Reactions were stopped by taking a 3 μl aliquot from the master mix and mixing it with three volumes of gel loading buffer (80% formamide, 20 mM EDTA). Products were analyzed via 12% denaturing PAGE and visualized using a Phosphorimager. Band intensities were quantified with the Quantity One software from BioRad, mean values and standard deviation were calculated from three independent experiments.

### Enzyme kinetics

Steady-state kinetics of KOD pol (wildtype and R501C) were measured under single completed hit conditions [56–58]. Single-nucleotide incorporation primer extension experiments were carried out followed by analysis via denaturing polyacrylamide gel electrophoresis (PAGE) and visualization by autoradiography. KOD pol concentrations were chosen so that no more than 20% of the primer was extended. The incorporation rates were determined at various dGTP concentrations over a time course ranging from 0–120 seconds. The percentage of extended primer was plotted against incubation time for each dNTP concentration and reaction velocities were determined by multiplying with primer concentration. Mean values and standard deviations were calculated from three independent experiments. For kinetic analysis according to Michaelis-Menten, mean values of reaction velocities were divided by KOD pol concentrations and plotted against dNTP concentrations. The experimental data were fitted to a hyperbolic equation with Origin2015 in order to determine K_M_ and k_cat_.

### Real time PCR using human genomic DNA

Reactions mixtures (10 μl) contained 120 mM TrisHCl pH 8, 10 mM KCl, 6 mM (NH_4_)_2_SO_4_, 0.1% Triton, 1.5 mM MgCl_2_, 1x SYBR Green I, 5% DMSO, 10 ng genomic DNA and 150 nM of the respective KOD DNA polymerase mutant. For amplification 100 nM of each primer were used; the forward primer ending in a C at its 3’ end which was either matched or mismatched with the F2G or F2A template respectively (5’-d(CTG GGA GCA TTG AGG CTC)-3’), reverse primer (5’-d(CCG CCT GAA GAA GTG GAT AC)-3’). Real time PCR experiments had an initial denaturation at 95°C for 3 min followed by amplification over 40 cycles with denaturation at 95°C for 15 s, annealing at 65°C for 15 s and elongation at 72°C for 45 s. Data was collected from at least three independent experiments. Melting curves were measured immediately after PCR amplification. PCR products (163 bp) were analyzed by 2.5% agarose gel electrophoresis.

## Results

### Site specific library generation

**Fig 1:**
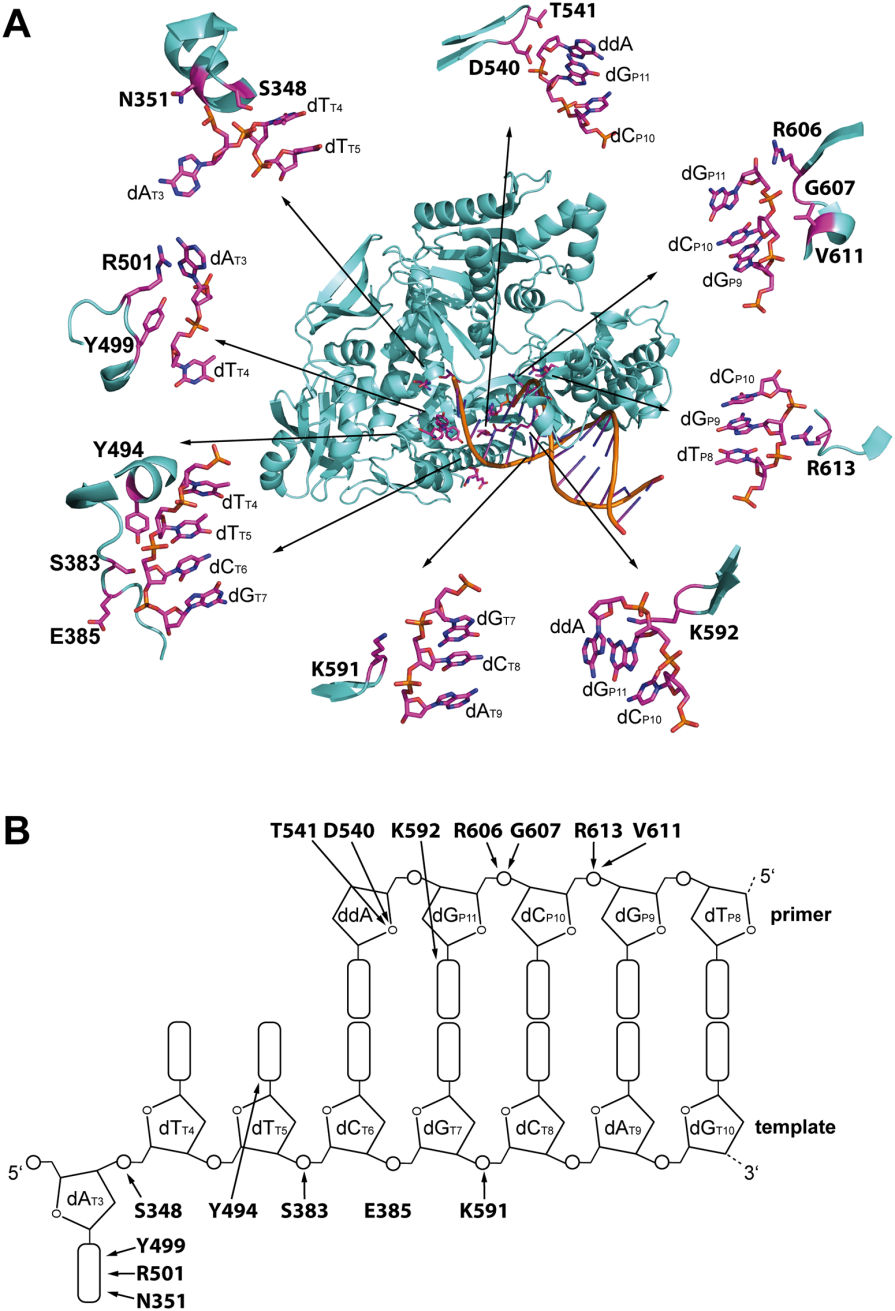
Mutation sites in KOD pol. (A) Crystal structure of a binary complex of KOD pol (PDB code 4K8Z [48]. The sections shown in detail, illustrate how the investigated amino acids interact with the primer or template backbone. (B) Schematic drawing of the primer-template duplex. Amino acids and their respective interactions with the DNA substrate are indicated by arrows according to [48].

In order to identify amino acid residues, which might be crucial for KOD pol function and selectivity, we used a structure based approach. Fig 1 shows a crystal structure of a binary complex of KOD pol bound to a DNA substrate [48]. Residues for mutation were chosen by their proximity with either primer or template DNA during the catalyzed reaction, as seen in Fig 1. Similar studies on sequence family A *Thermus aquaticus* DNA polymerase focused solely on interactions along the primer backbone [37]. However, our study also includes amino acids which contact the template strand. Saturation mutagenesis was performed via 19 defined PCR reactions per codon, in order to gain all possible amino acid exchanges at each targeted site.

### Screening of the libraries for PCR activity

**Fig 2:**
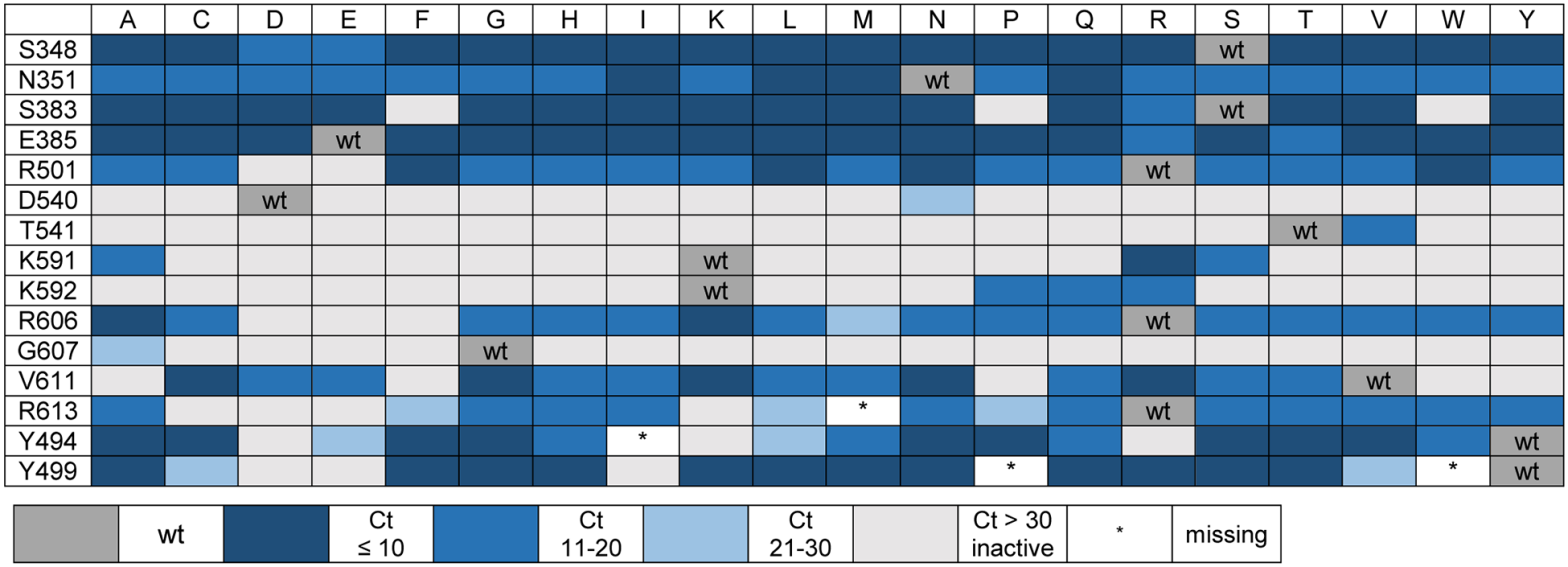
Results of screening the KOD pol library as activity chart. Amino acids of interest are given on the left, amino acid exchanges are depicted at the top in single letter code. Colors indicate activity of mutant proteins measured as Ct values in real time PCR screening assays using bacterial lysates. Darker shades of blue indicate lower Ct values, grey shows PCR inactive mutants. Boxes marked with asterisks show variants that could not be obtained during several rounds of cloning.

After cloning, the KOD pol libraries were expressed, protein production was checked by SDS gel analysis to ensure little variations in protein levels and heat-treated bacterial lysates were screened for PCR activity. For this, a fragment of the human NANOG gene was amplified with corresponding primers [51] and DNA double strands were visualized in real time PCR reactions utilizing SYBR Green I. The resulting amplification curves gave direct evidence whether a given mutation resulted in a PCR-active or -inactive enzyme variant. Analysis of threshold-cycle values (Ct values) provided insights about the degree of activity of each generated enzyme—low Ct values indicating higher activity than higher Ct values. Fig 2 depicts a semi-quantitative summary of the screening process, color coded and grouped into Ct ranges observed in qPCR reactions from two independently generated library lysates.

Amplification curves with wildtype KOD pol showed low Ct values in each case and reactions with mutant enzymes were compared and grouped accordingly. As seen in Fig 2, amino acids D540, T541, K591, K592 and G607 are crucial for KOD pol PCR activity, as almost all amino acid substitutions at these sites result in inactive enzymes. All exchanges of amino acids S348, N351, E385 resulted in enzyme variants with near wildtype levels of PCR activity. Mutations at the remaining sites gave a mix of equally active, less active as well as inactive DNA polymerases. Four KOD pol variants, namely R613M, Y494I, Y499P and Y499W could not be obtained even after several rounds of cloning. As further experiments showed, these sites turned out to be less interesting within our study, so no further efforts were made to obtain these mutated enzymes.

### Screening for increased mismatch extension selectivity

**Fig 3:**
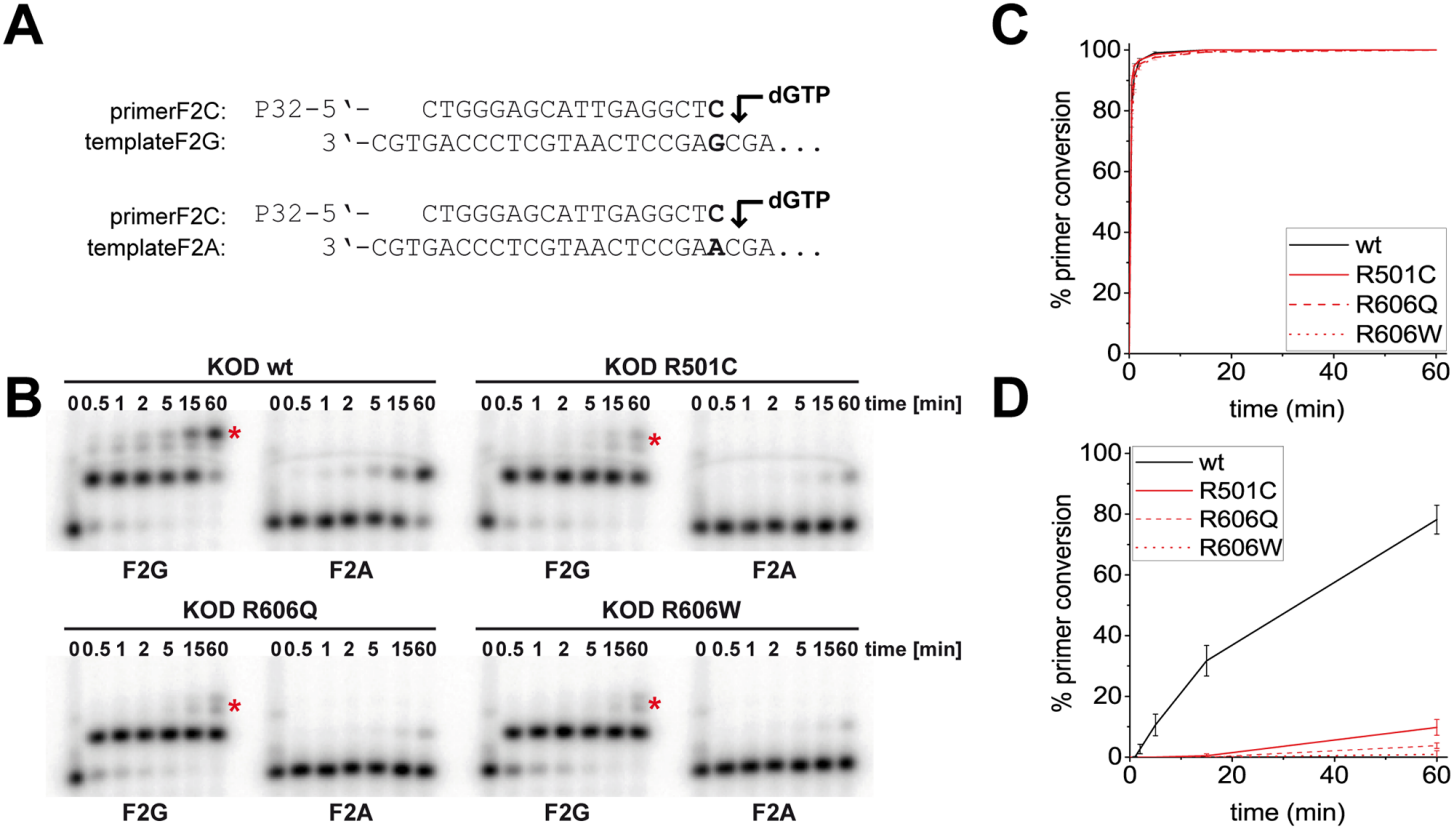
Single nucleotide primer extension experiments using KOD pol wildtype and mutants. (A) primer and (partial) template sequence from the prothrombin factor II gene. The 3’ end of the radioactively labeled primer was either matched or mismatched to the template. (B) PAGE analysis of single nucleotide extension of the primer by either KOD pol wildtype or mutants, as indicated. Reactions were carried out for the indicated times. (C) + (D) percentage of primer conversion vs time for match and mismatch case, respectively. Gel bands were quantified with the Quantity One software from BioRad, results are shown as means +/- SD of three independent experiments. Graphs were prepared with Origin2015.

Lysates of active polymerase variants were next screened for an altered ability to amplify from mismatched primer-template duplexes. Therefore, the same DNA fragment as in the activity screening described above was used. However, instead of the matched forward primer, three further forward primers were used, differing in the base at their 3’ end. This resulted in four different reactions: a matched primer-template duplex terminating in a correct C-G Watson-Crick base pairing and three cases of mismatched primer-template duplexes, terminating in either C-A, C-T or C-C. Three variants with single amino acid exchanges emerged as especially interesting from this screen, by showing very large Ct differences between matched and mismatched primers, indicating a high mismatch extension selectivity: R501C, R606Q and R606W (S1 Fig). These three were further characterized in single nucleotide primer extension reactions, using a DNA sequence from the prothrombin factor II carrying a known single nucleotide polymorphism (SNP): the prothrombin G20210A variation is a marker of predisposition for venous thromboembolism and one of the most common genetic polymorphisms [59]. We used a 5’-radioactively labeled primer terminating in a cytosine and two templates carrying either a G or an A opposite the 3’ primer terminus (Fig 3A). Reaction mixtures containing primer, template and the respective enzyme were denatured, re-annealed and primer extension was started by addition of dGTP. The reactions were incubated at a constant temperature of 55°C and stopped after six different time points (see Fig 3B), followed by analysis via denaturing PAGE and visualization by autoradiography. As depicted in Fig 3B and 3C, KOD pol wildtype as well as the enzyme variants extended almost all of the primer under these conditions. Also, all KOD pol variants perform a misincorporation after prolonged incubation, when employing the G-template (match case) (Fig 3B, marked by a red asterisk). KOD pol wildtype is able to convert a significant portion of the primer even when the template is mismatched with the primer (Fig 3B and 3D).

However, less than 80% of the primer is extended in this case, showing an intrinsic mismatch discrimination of the KOD pol. Notably, all three variants fail to extend the primer significantly when it is paired in a mismatched primer-template duplex, with less than 10% primer conversion being detected through analysis of PAGE gels. Clearly, the single amino acid exchanges lead to KOD pol variants with significantly increased mismatch extension selectivity in comparison to the wildtype.

To further evaluate the mismatch extension selectivity, we determined steady-state kinetics [56–58] for dGMP incorporation after either the matched (C-G) or mismatched (C-A) primer-template duplex for KOD pol wildtype and KOD R501C which still shows detectable mismatch extension (Table 1 + S2 Fig). T In case of the wildtype enzyme the mismatch extension selectivity stems from significant changes in K_M_ (approx. 1047-fold increase in K_M_ for mismatch) while in KOD R501C the K_M_ increase is only approx. 4-fold. However, in KOD R501C the k_cat_ for the mismatched reaction is approx. 250-fold decreased in comparison to approx. 4-fold in the wildtype enzyme. However, in typical PCR reactions, 200 μM dNTPs are required that are nearly saturating all reactions in terms of nucleotide substrate binding and thus, the differences in k_cat_ are more prevalent resulting in the observed degree of discrimination in the real time PCR experiments and primer extensions.

**Table 1:** Analysis of steady-state kinetics of dGMP incorporation after either matched or mismatched primer-template substrate.

enzyme	primer-template	K_M_ [μM]	k_cat_ [s^-1^]	k_cat_/K_M_ [s^-1^ μM^-1^]
wt	C-G (match)	0.168 ± 0.04	0.81 ± 0.12	4.8 ± 1.9
	C-A (mismatch)	176 ± 59	0.23 ± 0.04	0.001 ± 7 E-4
R501C	C-G (match)	96 ± 21	8.3 ± 0.6	0.09 ± 0.02
	C-A (mismatch)	391 ± 129	0.033 ± 0.005	8 E-4 ± 4 E-5

### Real time PCR analysis of the identified variants

**Fig 4:**
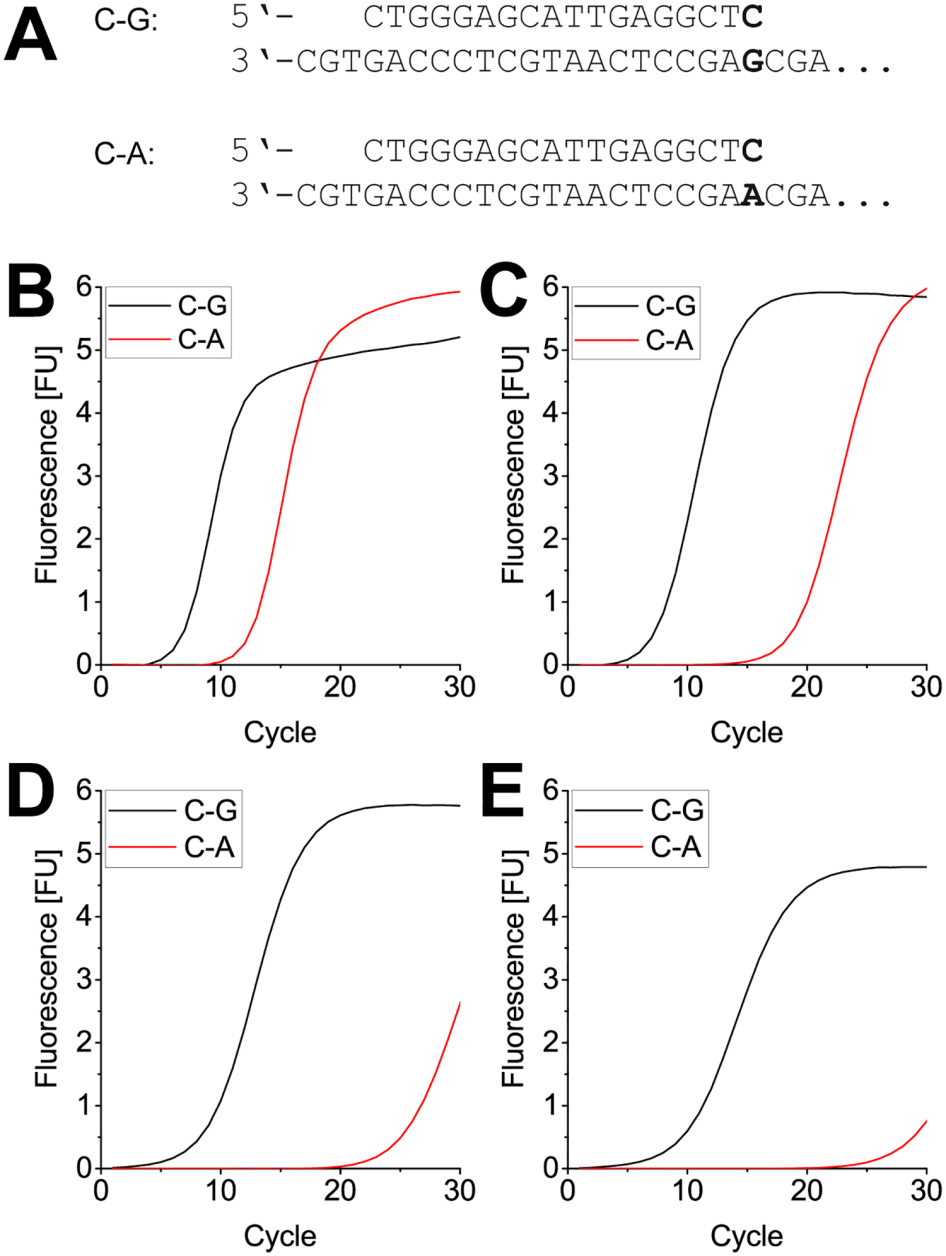
Real time PCR of a synthetic prothrombin Factor II oligonucleotide with KOD wildtype and mutants. (A) primer and (partial) template sequence from the prothrombin Factor II gene. The 3’ end of the primer was either matched or mismatched to the template. (B) real time PCR curves obtained when using KOD pol wildtype. The enzyme distinguishes only little when amplifying from matched (C-G) or mismatched (C-A) primer-template duplex. (C) + (D) + (E) real time PCR curves obtained by using KOD R501C, KOD R606Q and KOD R606W respectively. Increased discrimination is shown, as indicated by slower PCR amplification from mismatched (C-A) primer-template duplexes. R501C barely reaches saturation after 30 cycles; both R606 variants are almost inactive in the mismatch case under the applied conditions.

In order to further test the identified variants, we also performed qPCR with the prothrombin factor II DNA. Fig 4 shows the qPCR amplification curves for KOD pol wildtype and the variants, with the black curve representing amplification from the matched primer-template duplex and the red curve representing amplification from the mismatched primer-template duplex.

**Fig 5:**
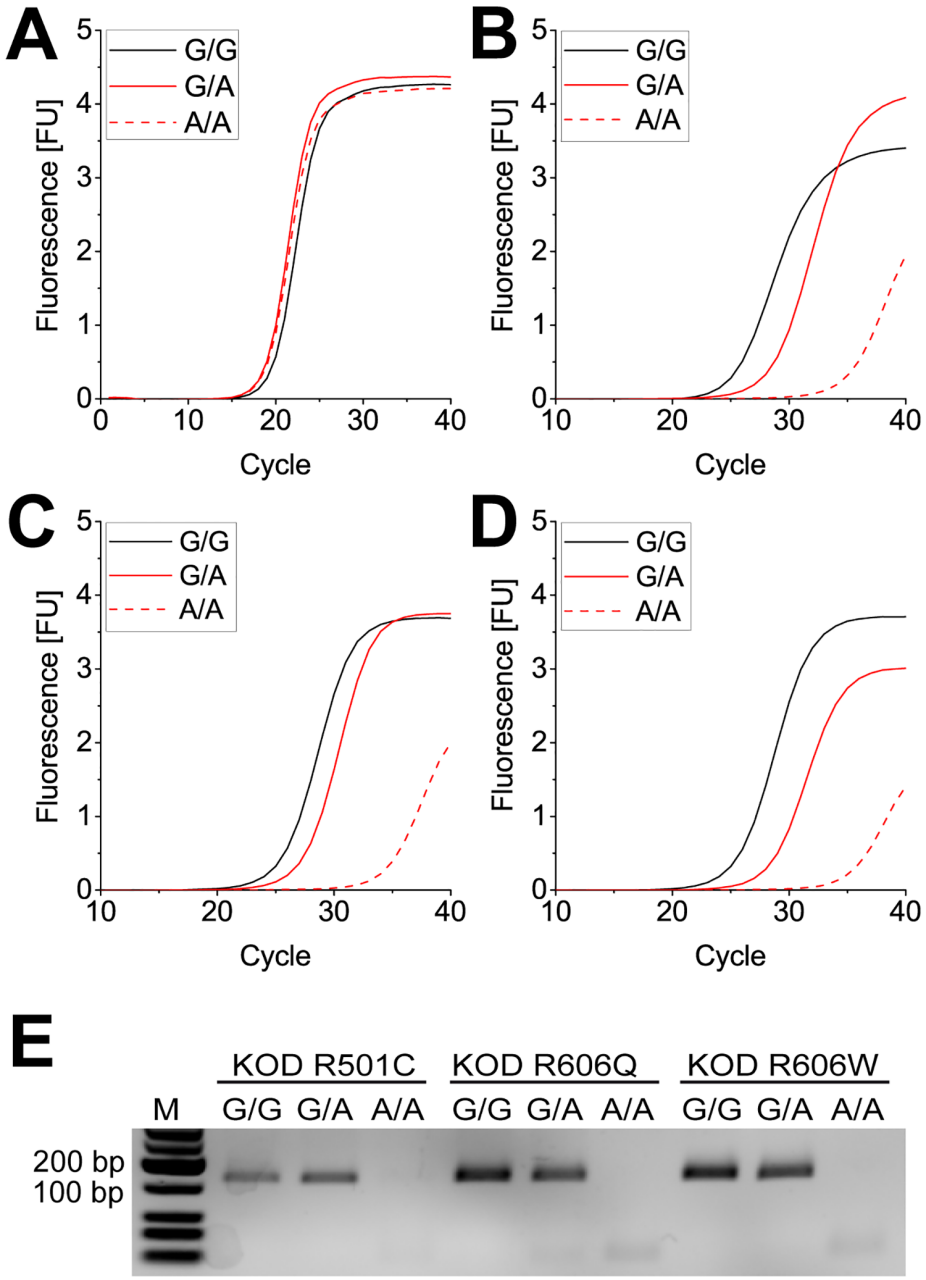
Real time PCR of prothrombin factor II Human gDNA with KOD pol wildtype and mutants. Real time PCR curves when using a forward primer that terminates opposite the SNP site in either a matched or mismatched fashion, depending on the template. (A) real time PCR curves obtained when using KOD pol wildtype. No discrimination is seen for either of the DNA samples: G-wt (genotype G/G), A-heterozygous (genotype G/A) or A-homozygous (genotype A/A). (B)+(C)+(D) real time PCR curves when using KOD pols R501C, R606Q, and R606W, respectively. Discrimination is seen for the amplification of the A-homozygous (A/A) Factor II template which is severely slowed down when using the KOD mutants. (E) agarose gel electrophoresis analyzing PCR products from (B)-(D). M = marker. PCR products are only visible in samples containing template DNA with at least one allele that results in a matched primer-template duplex (C-primer with G/G and G/A DNA template); the homozygous A/A template does not yield a PCR product.

Again the intrinsic discrimination ability of the KOD pol wildtype can be seen (Fig 4B), with a ΔCt of ~5. This difference in Ct values is increased to ~10 for KOD pol R501C (Fig 4C) and much larger even for KOD pols R606Q and R606W with ΔCt ~15 and ~20, respectively (Fig 4D and 4E). To further verify our findings and as a proof of concept that a DNA polymerase with increased mismatch extension selectivity can directly detect SNPs, we additionally performed qPCR with human HeLa genomic reference DNA (WHO International Standard) for the prothrombin factor II G-to-A variation. All mutants exhibit an activity comparable to KOD pol wildtype, with Ct values around 20 for amplification in the match case (Fig 5A–5D, black curves).

The assay also covered both heterozygous and homozygous allelic sample DNA. Interestingly, KOD pol wildtype was not able to show discrimination when amplification was performed from genomic DNA, as all three reactions exhibited identical Ct values, showing undistinguishable qPCR curves (Fig 5A). Notably, all three variants showed already a small discrimination between wildtype genotype G/G and heterozygous genotype G/A (Fig 5B–5D, red solid line). When employing the homozygous genotype A/A DNA, all three KOD pol variants exhibited a high mismatch extension selectivity, visualized by a ΔCt of ~10 cycles compared to the wildtype genotype (Fig 5B–5D). Furthermore, as shown in Fig 5E, specific product formation was only detected when at least one allelic copy resulted in a matched primer-template duplex (C-primer with G/G or G/A template DNA); no specific product formation was observed with the mismatched homozygous A/A template DNA.

### Action of the variants on 5-methyl cytosine

**Fig 6:**
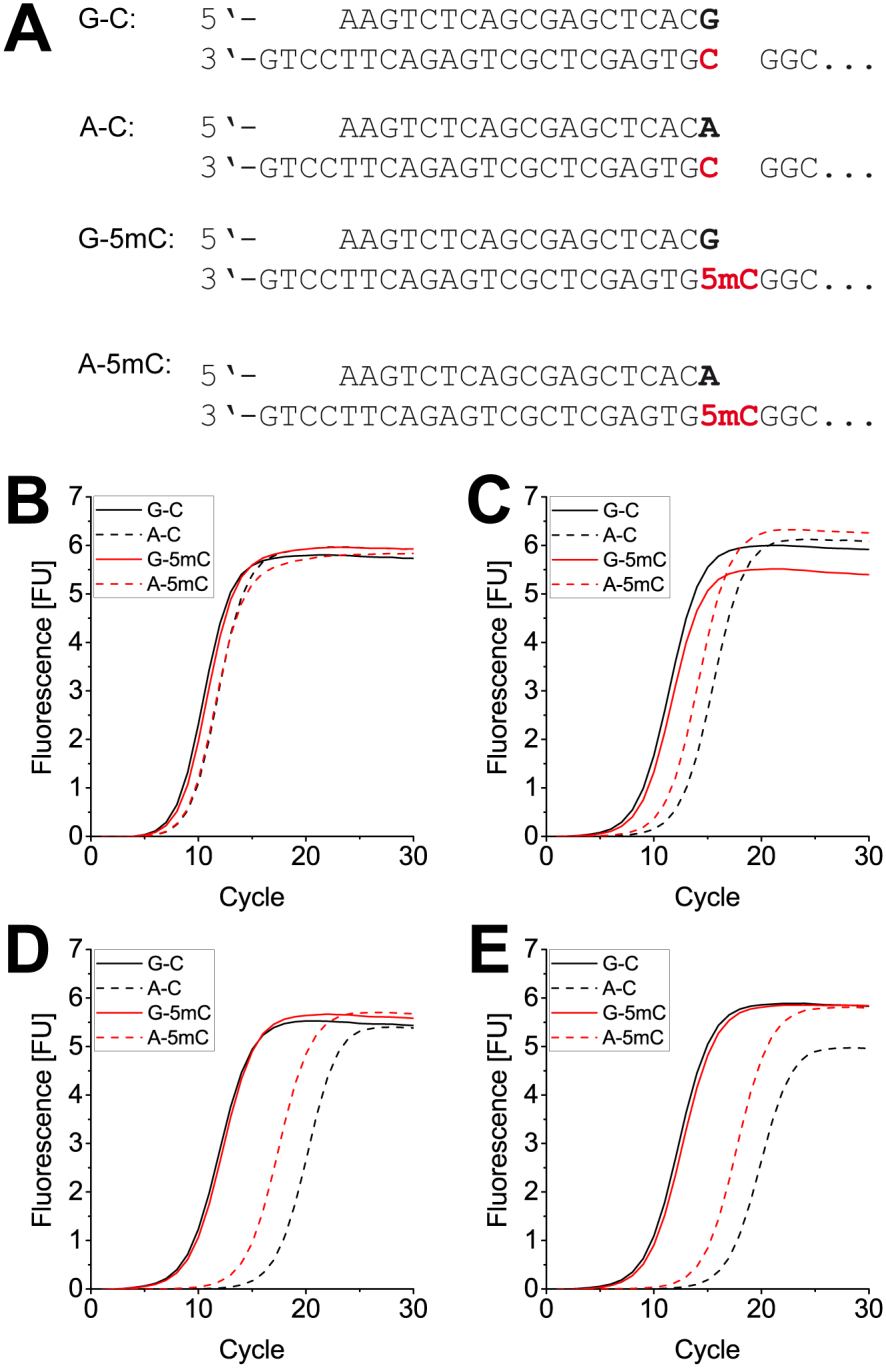
Real time PCR for cytosine methylation detection. (A) The chosen forward primer terminates opposite either C or 5mC, depending on the template. Two forward primers were used, carrying either G or A at their 3’ end, resulting in matched or mismatched primer-template duplex respectively. (B) real time PCR curves using KOD pol wildtype. Equal amplification is observed for all four primer/template samples. (C) real time PCR curves using KOD pol R501C. Amplification from the matched primer is not distinguishable between C or 5mC. Amplification from the mismatched primer is in both cases slightly slower, but not significantly different between C and 5mC. (D) + (E) real time PCR curves using KOD pol R606Q and KOD pol R606W, respectively. Matched primer-template duplexes are amplified without discrimination and comparable to KOD pol wildtype. In the mismatch case, methylated DNA is clearly favored, demonstrating the ability of the enzyme variants to discriminate between C and 5mC.

As has been shown before, DNA polymerases are able to distinguish between cytosine (C) and 5-methyl cytosine (5mC), the most important epigenetic mark in DNA [60], when a mismatch at the primer end is formed opposite the C or 5mC in the template [49]. Since the KOD pol variants identified in the present study showed high mismatch discrimination abilities, we were encouraged to test whether they were also able to detect 5mC in a similar fashion as the previous study. We performed qPCR assays in the sequence context of the BRCA1 gene. Hypermethylation of the BRCA1 promoter region is associated with breast and ovarian cancer [55] and analysis of the methylation status of this region is therefore of particular interest. We employed two templates that only differ at the position directly opposite the 3’ terminus of the primer, carrying either a C or a 5mC (see Fig 6A). It is shown in Fig 6B that KOD pol wildtype does not discriminate between C and 5mC in both matched and mismatched amplification, as all four amplification curves show near identical Ct values. However, all three KOD pol variants exhibit some discrimination abilities when amplifying from a mismatched primer-template duplex (A-C and A-5mC). The delay of ~5 cycles from the mismatch extension is again seen for KOD pols R501C, R606Q and R606W with an additional delay of 2–3 cycles when the mismatch is opposite an unmethylated vs. a methylated cytosine (Fig 6C–6E, compare black vs red dashed lines).

## Discussion

Here we reported on the design and evaluation of KOD pol libraries that were developed in order to find KOD pol variants with increased mismatch extension selectivity. Residues were selected for saturation mutagenesis due to their proximity to either the primer or template strand. We generated extensive data on the activity of single amino acid exchange variants, showing that some residues are crucial for the retention of PCR activity, whereas other residues can be substituted while retaining PCR activity. We employed the generated libraries in screening studies, looking for variants with increased primer-template mismatch discrimination selectivity and were able to successfully identify three KOD pol variants that bear single site substitutions at sites R501C, R606Q and R606W, respectively. These three variants were characterized in primer extension studies as well as qPCR assays. Our data clearly depict the ability of KOD pol wildtype as well as the three variants to efficiently convert the primer, when paired with a correct Watson-Crick matched template strand. While the wildtype enzyme was able to extend a mismatched primer, the three investigated mutants were much less efficient. The same behavior was observed in a qPCR assay, showing strongly delayed amplification from mismatched primer-template DNA by the identified mutants. Furthermore, we showed the mismatch extension selectivity when employing human genomic HeLa DNA. Efficient DNA amplification was only observed for the match case; significantly less efficient product formation was observed for all three mutants in the mismatch case.

The sites at which amino acid exchanges led to increased KOD pol selectivity contact the primer (R606) as well as the template (R501) (see Fig 1). Our previous studies based on rational design, identified solely residues that interact with the primer 3’ end as crucial for tuning selectivity [37]. Our approach in the presented study also included residues which contact both primer and template at the active site as well as distal from the active site. Arginine 606 interacts with the primer phosphate backbone between the -2 and -3 nucleotide position (Fig 1B) and as our study clearly shows, changes to this region can heavily influence enzyme selectivity. As mentioned, we also investigated amino acids contacting the template strand. Indeed, one of the identified KOD pol variants carried an amino acid exchange at position R501, which contacts the template +2 nucleobase (Fig 1B). Since this enzyme variant also exhibits increased mismatch extension selectivity, this highlights the importance of DNA polymerase-template interactions that are distal from the active site for selectivity.

Substituting the arginine residues with cysteine, glutamine and tryptophan removes positive charges from the polymerase that likely stabilize the interactions with the primer-template duplex regardless if matched or mismatched. The loss of potential electrostatic interactions and a less polar environment was reported to increase mismatch extension selectivity [35, 37, 44, 61] and our findings here are in agreement with this observation.

We further showed that the mismatch sensitive mutants are also suitable for the qPCR based discrimination of methylated cytosine. While matched primer-template duplexes could not trigger any distinction between C and 5mC opposite the primer 3’ terminus, qPCR reactions with mismatched primer-template duplexes showed delayed amplification when the mutated KOD pols were employed. It is not surprising that there is no discrimination in the match case, as the methyl group of 5mC does not directly interfere with Watson-Crick base pairing and thus should not significantly alter substrate binding energies. Along these lines, one would expect to find no difference between C and 5mC when both are opposite the same mismatched primer 3’ terminus. Shen et al., previously investigated the mismatch extension properties opposite C and 5mC [62]. Significant differences in fidelity were observed with AMV reverse transcriptase, but not with any of the investigated family A or family B DNA polymerases. Three previous studies from our group [37, 49, 51] as well as the presented work clearly show the influence of cytosine methylation on DNA polymerase fidelity. The addition of the methyl group might alter substrate recognition of the enzyme, thus changing the mismatch extension properties. Obviously, for the KOD pol wildtype this difference is not large enough for an observable C vs 5mC discrimination. However, substitution of a single amino acid seems to be able to facilitate a high enough energy difference, resulting in the enzyme variants displaying an increased selectivity in the presence of methylated DNA.

In summary, we have identified three amino acid exchanges in KOD pol, which retained high activity, while exhibiting a distinct mismatch extension selectivity. Our study shows the potential that lies within the combination of rational design and a stringent screening approach in order to identify engineered DNA polymerases with enhanced fidelity and potential biotechnological and diagnostic application possibilities.

## Supporting information

S1 Fig Data from initial qPCR screening of KOD pol variants. (A) qPCR curves obtained when using all generated variants at position R501. Black solid lines represent reactions with a matched C-G primer-template duplex. Red solid, red dashed and red dotted lines represent C-A/C-T/C-C mismatches respectively. Variant R501C (top row, third panel from the left) showed the most pronounced discrimination between matched and all three mismatched primer-template duplexes and was therefore used for further characterization. (B) qPCR curves obtained when using all generated variants at position R606. Black solid lines represent reactions with a matched C-G primer-template duplex. Red solid, red dashed and red dotted lines represent C-A/C-T/C-C mismatches respectively. Variant R606L (third row, third panel from the left) showed pronounced discrimination during initial screening experiments employing bacterial lysates, which could not be reproduced with the purified enzyme. Variants R606Q (fourth row, third panel from the left) and R606W (fifth row, third panel from the left) showed clear discrimination between matched and all three mismatched primer-template duplexes and were used for further characterization.
(PDF)

S2 Fig Steady state kinetics of dGMP incorporation after matched or mismatched primer-template duplex substrate. (A) KOD pol wt with matched primer-template duplex (C-primer, G-template). (B) KOD pol wt with mismatched primer-template duplex (C-primer, A-template). (C) KOD pol R501C with matched primer-template (C-primer, G-template). (D) KOD pol R501C with mismatched primer-template duplex (C-primer, A-template).
(PDF)
