# Supplementary material for: Variants of sequence family B Thermococcus kodakaraensis DNA polymerase with increased mismatch extension selectivity
Source: PLoS One. 2017 Aug 23;12(8):e0183623. doi: 10.1371/journal.pone.0183623 (PMC5568139; doi:10.1371/journal.pone.0183623)
Supplement: S1 Fig — (A) qPCR curves obtained when using all generated variants at position R501. Black solid lines represent reactions with a matched C-G primer-template duplex. Red solid, red dashed and red dotted lines represent C-A/C-T/C-C mismatches respectively. Variant R501C (top row, third panel from the left) showed the most pronounced discrimination between matched and all three mismatched primer-template duplexes and was therefore used for further characterization. (B) qPCR curves obtained when using all generated variants at position R606. Black solid lines represent reactions with a matched C-G primer-template duplex. Red solid, red dashed and red dotted lines represent C-A/C-T/C-C mismatches respectively. Variant R606L (third row, third panel from the left) showed pronounced discrimination during initial screening experiments employing bacterial lysates, which could not be reproduced with the purified enzyme. Variants R606Q (fourth row, third panel from the left) and R606W (fifth row, third panel from the left) showed clear discrimination between matched and all three mismatched primer-template duplexes and were used for further characterization. (PDF) [file pone.0183623.s001.pdf]

A

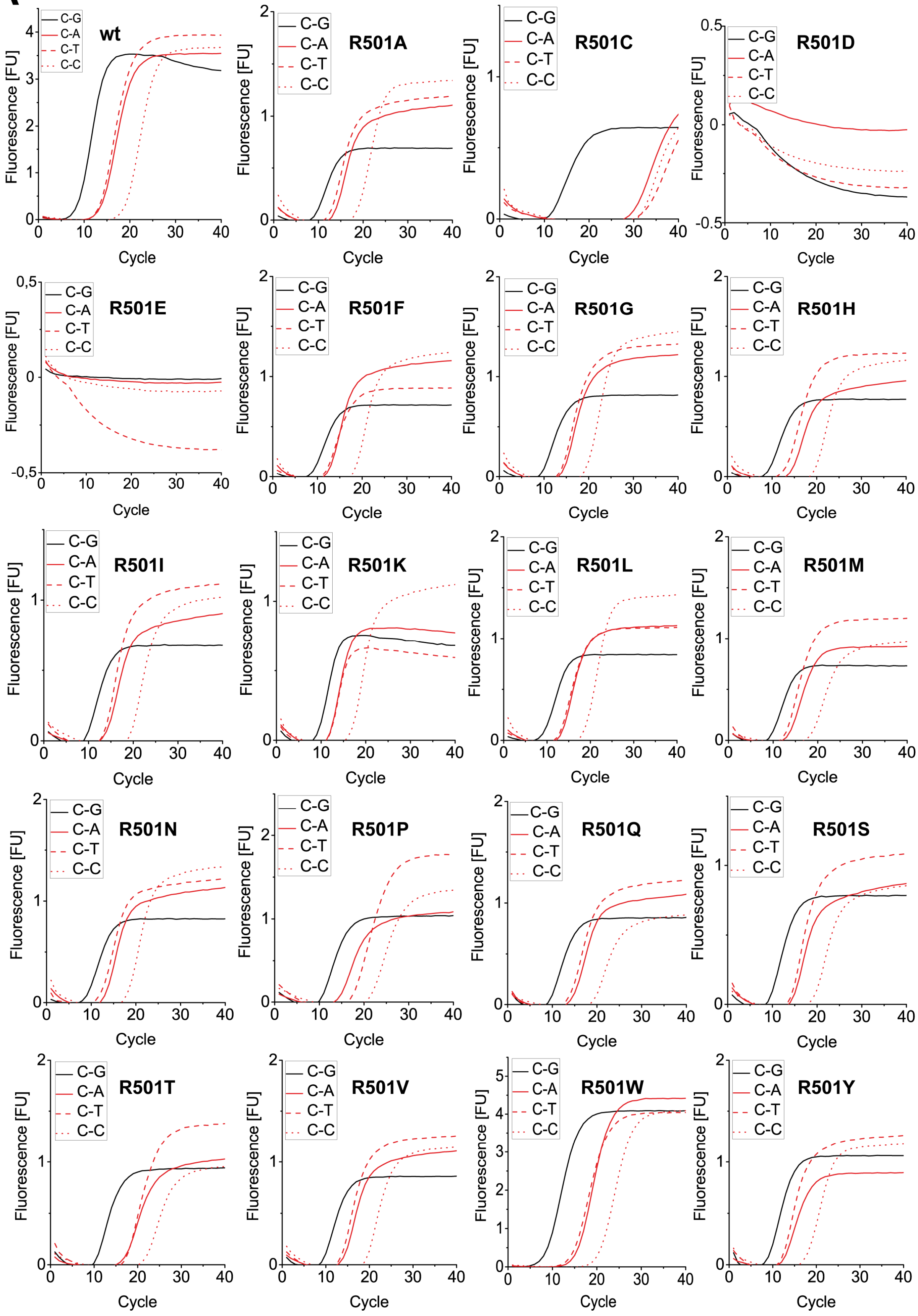

wt
R501A
R501C
R501D
R501E
R501F
R501G
R501H
R501I
R501K
R501L
R501M
R501N
R501P
R501Q
R501S
R501T
R501V
R501W
R501Y
C-G
C-A
C-T
C-C
Fluorescence [FU]
Cycle

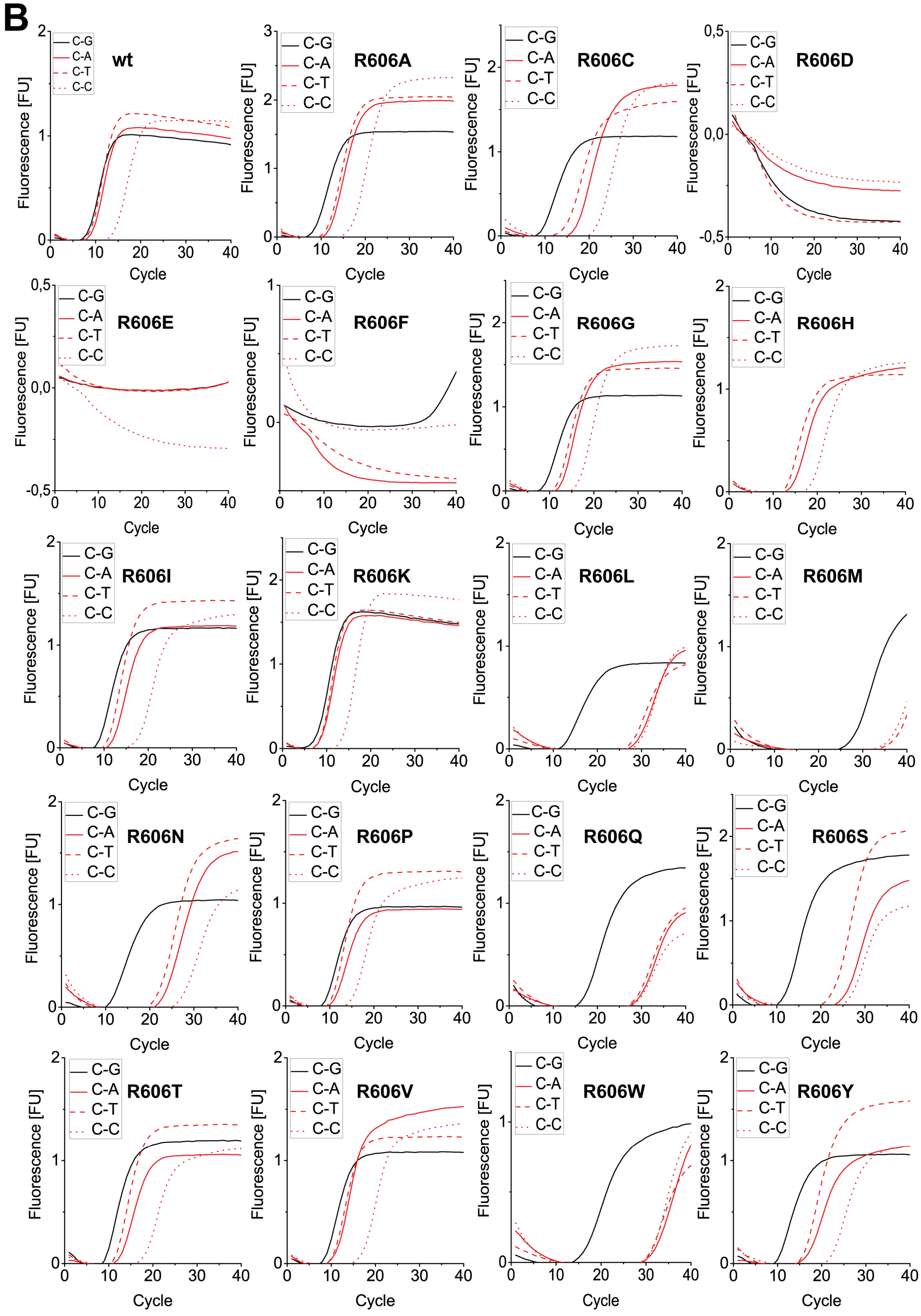

S1 Fig. Data from initial qPCR screening of KOD pol variants.

(A) qPCR curves obtained when using all generated variants at position R501. Black solid lines represent reactions with a matched C-G primer-template duplex. Red solid, red dashed and red dotted lines represent C-A/C-T/C-C mismatches respectively. Variant R501C (top row, third panel from the left) showed the most pronounced discrimination between matched and all three mismatched primer-template duplexes and was therefore used for further characterization.

(B) qPCR curves obtained when using all generated variants at position R606. Black solid lines represent reactions with a matched C-G primer-template duplex. Red solid, red dashed and red dotted lines represent C-A/C-T/C-C mismatches respectively. Variant R606L (third row, third panel from the left) showed pronounced discrimination during initial screening experiments employing bacterial lysates, which could not be reproduced with the purified enzyme. Variants R606Q (fourth row, third panel from the left) and R606W (fifth row, third panel from the left) showed clear discrimination between matched and all three mismatched primer-template duplexes and were used for further characterization.
