# Supplementary material for: Variants of sequence family B Thermococcus kodakaraensis DNA polymerase with increased mismatch extension selectivity
Source: PLoS One. 2017 Aug 23;12(8):e0183623. doi: 10.1371/journal.pone.0183623 (PMC5568139; doi:10.1371/journal.pone.0183623)
Supplement: S2 Fig — (A) KOD pol wt with matched primer-template duplex (C-primer, G-template). (B) KOD pol wt with mismatched primer-template duplex (C-primer, A-template). (C) KOD pol R501C with matched primer-template (C-primer, G-template). (D) KOD pol R501C with mismatched primer-template duplex (C-primer, A-template). (PDF) [file pone.0183623.s002.pdf]

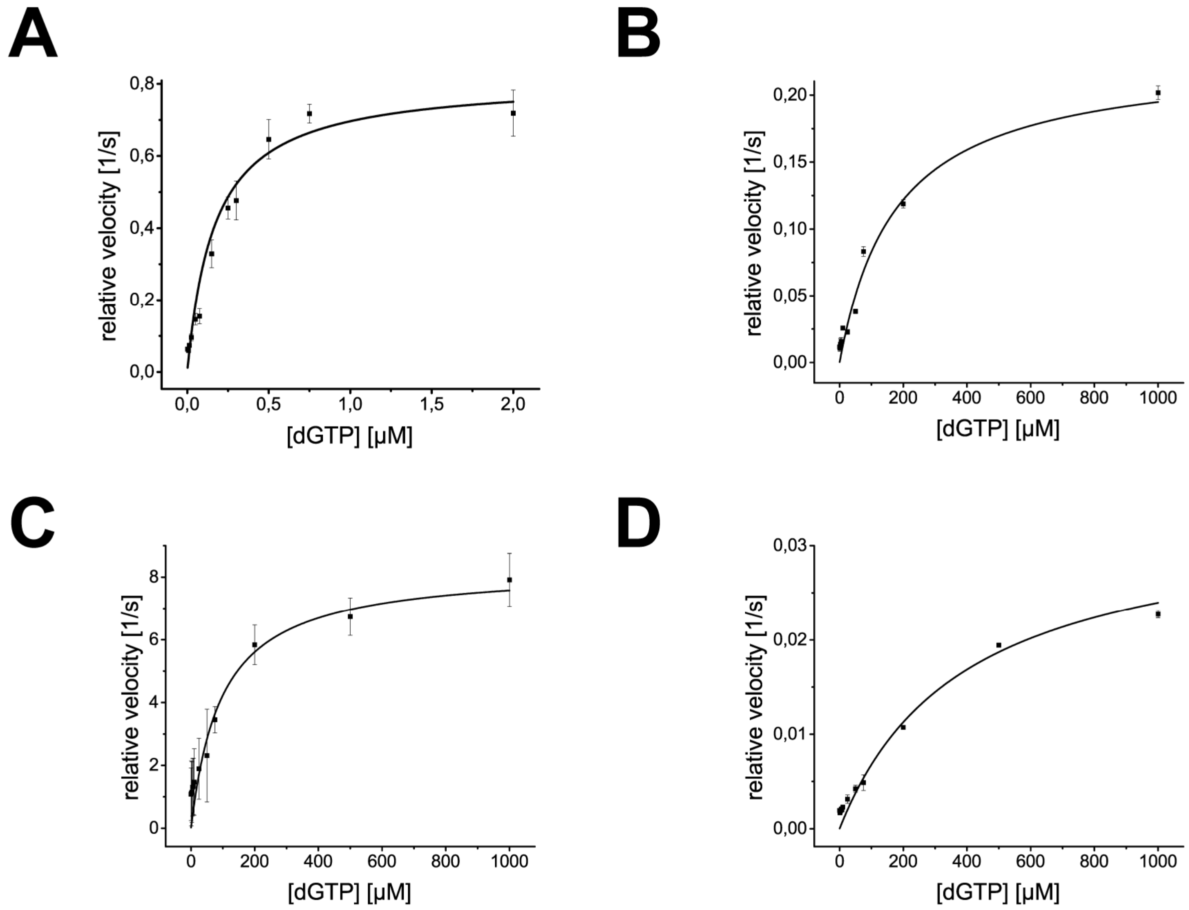

S2 Fig. Steady state kinetics of dGMP incorporation after matched or mismatched primer-template duplex substrate.

(A) KOD pol wt with matched primer-template duplex (C-primer, G-template). (B) KOD pol wt with mismatched primer-template duplex (C-primer, A-template). (C) KOD pol R501C with matched primer-template (C-primer, G-template). (D) KOD pol R501C with mismatched primer-template duplex (C-primer, A-template).
